# Ecology determines how low antibiotic concentration impacts community composition and horizontal transfer of resistance genes

**DOI:** 10.1038/s42003-018-0041-7

**Published:** 2018-04-19

**Authors:** Johannes Cairns, Lasse Ruokolainen, Jenni Hultman, Manu Tamminen, Marko Virta, Teppo Hiltunen

**Affiliations:** 1 Department of Microbiology, University of Helsinki, P.O. Box 56, 00014 Helsinki, Finland; 2 Department of Biosciences, University of Helsinki, P.O. Box 65, 00014 Helsinki, Finland; 3 Department of Aquatic Ecology, Eawag, Dubendorf, 8600 Zurich, Switzerland; 4 Department of Environmental Systems Science, ETH Zurich, 8092 Zurich, Switzerland

## Abstract

Low concentrations of antibiotics have numerous effects on bacteria. However, it is unknown whether ecological factors such as trophic interactions and spatial structuring influence the effects of low concentrations of antibiotics on multispecies microbial communities. Here, we address this question by investigating the effects of low antibiotic concentration on community composition and horizontal transfer of an antibiotic resistance plasmid in a 62-strain bacterial community in response to manipulation of the spatial environment and presence of predation. The strong effects of antibiotic treatment on community composition depend on the presence of predation and spatial structuring that have strong community effects on their own. Overall, we find plasmid transfer to diverse recipient taxa. Plasmid transfer is likely to occur to abundant strains, occurs to a higher number of strains in the presence of antibiotic, and also occurs to low-abundance strains in the presence of spatial structures. These results fill knowledge gaps concerning the effects of low antibiotic concentrations in complex ecological settings.

## Abstract (web summary)

Johannes Cairns et al. show that spatial structure and predation shape the ways in which a low concentration of antibiotics alters the composition and diversity of bacterial community. This study suggests that ecological factors influence how antibiotic resistance spreads.

## Introduction

The legacy of the use and misuse of antibiotics in recent decades has left us with a global public health crisis: antibiotic-resistant bacteria are on the rise, making it harder to treat infections^1^. Extensive, uncontrolled use of antibiotics can result in the presence of low, sub-inhibitory concentrations (hereafter, sub-minimal inhibiting concentrations, or sub-MICs) in the environment and the guts and tissues of treated humans and animals, which might contribute to this problem. As well as selecting for antibiotic resistance mutations, sub-MICs can increase genetic and phenotypic variability and affect cell-to-cell signaling, biofilm formation, quorum sensing and gene expression in bacterial populations^2^. These effects might alter competitive interactions between species, and thereby, the structure and functioning of microbial communities. Notably, sub-MIC antibiotics can also reduce community diversity by increasing the variance in fitness among taxa^3–5^.

A major reason for the spread of antibiotic resistance is horizontal gene transfer, where mobile genetic elements, such as conjugative plasmids coding for antibiotic resistance, are exchanged between bacterial species^6^. Resistance plasmid harboring cells are predicted to be selected at antibiotic concentrations where the fitness of resistant cells exceeds the fitness of susceptible cells^7,8^. These concentrations can vary from MIC to levels several-hundred-fold lower, depending on how costly plasmid carriage is to cells^9^. However, positive selection can also inhibit plasmid transfer events by decreasing donor and/or recipient abundance or creating narrow conditions for transconjugant selection^9–11^, and high conjugative plasmid persistence has recently been reported in the absence of positive selection^12,13^. Because of these conflicting dynamics, the effect of sub-MICs on the spread of a conjugative plasmid introduced to a multispecies community is uncertain. Furthermore, there have been major methodological challenges in tracking plasmid transfer in complex communities^14–16^.

Key environmental factors, including species interactions and the level of spatial structuring of habitats, might play a critical role in how sub-MIC antibiotics influence bacterial community structure and horizontal transfer of antibiotic resistance genes. Competition for shared resources, predation, and parasitism represent fundamental ecological interactions shaping the structure of natural bacterial communities. Among these, predation by protozoa, which form the most abundant group of bacterivorous predators, is a well-documented driver of bacterial species diversity^17^. Community structure and diversity, in turn, mediate the spread of antibiotic resistance genes^18–21^. Predation might also directly affect the spread of resistance plasmids^12,14^. As well as experiencing complex biotic interactions, bacteria often reside in spatially structured environments, for example, on surfaces enabling the formation of multispecies biofilms. Spatial structuring is important for species composition^22^ and can promote horizontal gene transfer by sustaining high bacterial density and metabolic activity, and increasing physical proximity of bacterial cells^23–25^. Heightened conjugative plasmid transfer in biofilms has been shown in one- and two-species systems^26,27^, but not in more complex multispecies communities. Moreover, experimental demonstration linking community level effects of sub-MICs, spatial structuring, and horizontal transfer of antibiotic resistance genes is completely lacking.

Here we set out to investigate the interplay between sub-MIC antibiotics, trophic interactions and spatial structuring in determining community structure and horizontal transfer of a multidrug resistance plasmid in a multispecies bacterial community. We predicted altered community composition under different ecological scenarios. Specifically, antibiotics alone should result in decreased diversity, but this effect should decrease in the presence of predation, which maintains diversity, and spatial structures that confer bacteria increased antibiotic resistance. By changing the taxa present and their relative abundances these treatments should, in turn, result in different recipients of a broad host range conjugative plasmid. The number of plasmid recipient taxa may be decreased under sub-MIC antibiotic due to decreased diversity and/or inhibition of plasmid transfer events. Alternatively, selection for transconjugants might result in an increased number of plasmid recipient taxa, or the plasmid might spread to diverse taxa altogether independent of the presence positive selection. Finally, plasmid transfer should be altered in a spatially structured environment, either being promoted due to enhanced mating pair formation and host population colonization or being inhibited by spatial separation of donors and recipients.

## Results

### Ecology determines community effect of low antibiotic concentration

**Fig. 1:**
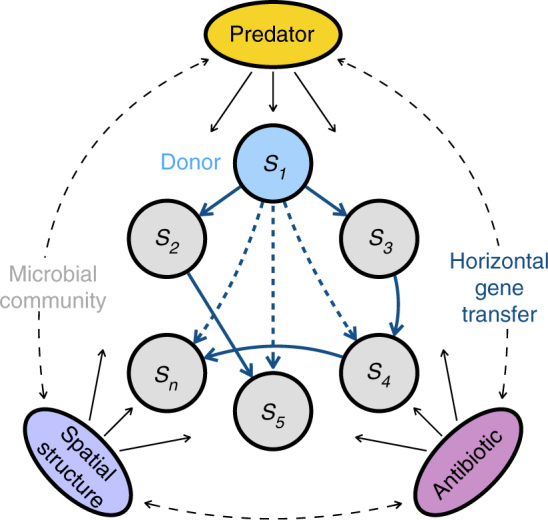
Conceptual representation of the study. Experimental bacterial community, containing 62 strains and RP4 plasmid only in donor *E. coli* at the beginning of the experiment, was exposed to the following manipulations (present/absent): spatial structure, ciliate predation and sub-MIC kanamycin concentration. During the 40-day microcosm experiment, we followed community dynamics, bacterial community structure, and spread of the plasmid (a result of horizontal gene transfer). The dashed lines denote potential interactions between the ecological factors or ecological factor-dependent plasmid transfer

To test for the effect of sub-MIC of an antibiotic (the aminoglycoside kanamycin) on community structure and horizontal spread of a conjugative antibiotic resistance plasmid, we performed a 40-day microcosm experiment with a 62-strain artificial bacterial community (Fig. 1). All communities included the plasmid donor strain *Escherichia coli* K-12 JE2571(RP4), harboring the multidrug resistance plasmid RP4. In a full factorial experimental design, we manipulated three key environmental factors (presence/absence): (1) sub-MIC antibiotic, represented by 2 µg ml^–1^ kanamycin (approximately 6% of mean MIC value of strains); (2) spatial structure (glass beads) in otherwise homogeneous microcosms; (3) an additional trophic level in the form of predation by the ciliated protozoan *Tetrahymena thermophila* CCAP 1630/1U. All treatments were replicated four times.

We determined bacterial community composition on day 8 (following 2 serial transfers of the bacterial community to fresh medium), day 24 (6 transfers), and day 40 (10 transfers; end-point) in the 40-day experiment, using 16S rRNA gene amplicon sequencing. Amplicons were mapped to a reference database created by Sanger-sequencing the near-full-length 16S rRNA gene sequence of experimental strains, allowing precise tracking of strains.

**Fig. 2:**
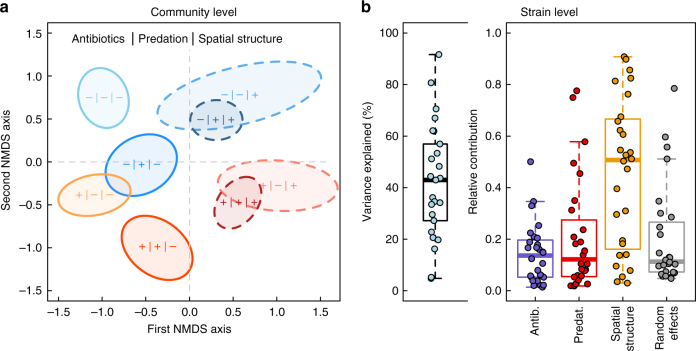
**a** Clustering of microbial communities (based on sample proximity in random forest classification) according to experimental design (*N* = 96). The +/– indicates the presence/absence of a particular treatment. Color codes for antibiotics (red/blue), color shade codes for predation (dark/light), and line style codes for spatial structure (continuous/dashed). **b** A hierarchical model of species communities (HMDS) of strain occurrence (strain traits account for 11% of variation in co-occurrence, after controlling for phylogenetic similarity) is able to capture about 20–90% (median 43%) of the variation (left panel), depending on the strains (low values around 5% are due to strain occurring in all samples; *N* = 96). Within the explained variation, strains also differ considerably in how they respond to the experimental treatments (right panel). Most variation is seen in the response to spatial structuring and this fraction tends to be negatively associated with the total fraction of variance explained (*ρ* = –0.38)

Firstly, analysis of community dissimilarity (based on multiple regression on distance matrices; MRM) revealed, in addition to significant temporal variation (*P* *<* 0.0001; for full results, see Supplementary Table 1 and Supplementary Fig. 1), a hierarchical influence of experimental treatments on community composition (Fig. 2a). The presence of kanamycin altered the community composition (*P* = 0.001), which was also affected by spatial structure (*P* < 0.001) and predation (*P* < 0.001). Furthermore, the effect of low antibiotic concentration depended on the presence of spatial structure and predation (antibiotic × spatial structure: *P* < 0.001; antibiotic × predation: *P* *=* 0.003; antibiotic × spatial structure × predation: *P* = 0.003) and the effect of predation depended on the presence of spatial structure (predation × spatial structure: *P* < 0.001). Together, the experimental factors (and their interactions) explained 70% of the variation in community dissimilarity. Compositional differences were due to both the variation in abundances and the presence/absence of taxa present in different communities (Supplementary Fig. 2).

Next, we combined the information of strain phylogenetic assignment, functional traits, and co-occurrence in a hierarchical model of species communities (HMSC)^28^ of the experimental set up. Together with strain traits (growth rate, MIC, and biofilm formation) and phylogenetic similarity (cophenetic correlation), the experimental treatments and random effects (sample + temporal level) captured on average 43% of variation in strain co-occurrence (Fig. 2b). The trait by treatment interaction estimation from HMSC indicated that there is a positive association between antibiotic MIC values and growth rate, and spatial structuring (Supplementary Table 2). When accounting for strain abundances, the MIC value tends to be positively associated with all treatments and growth rate with the antibiotic treatment in addition to spatial structuring (Supplementary Table 2).

**Fig. 3:**
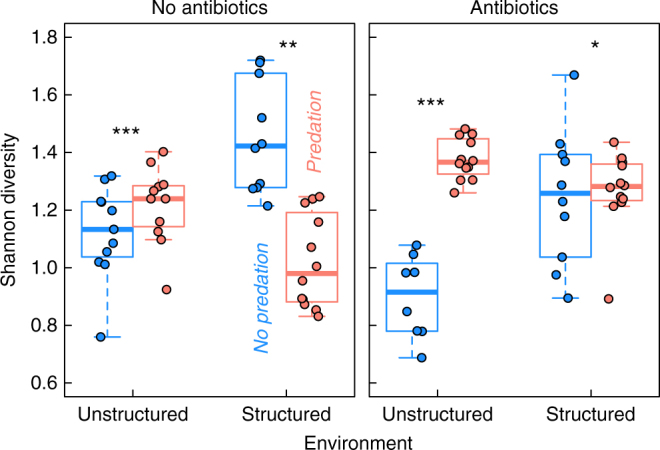
The response of species diversity (Shannon’s index) to predation and spatial structuring depends on whether antibiotic is present or not (*N* = 96). Antibiotic treatment (*P* < 0.0001) and spatial structure (*P* < 0.0001) both have a significant interaction with predation. Diversity was tested with a gls-model assuming group-specific residual variance

Finally, community diversity was quantified using the Shannon entropy (diversity of first degree), which accounts for both the abundance and evenness of strains present in the community. While the antibiotic treatment had an effect on diversity (*P* = 0.0001), the direction of the effect depended on the presence of spatial structure (interaction: *P* < 0.0001) (Fig. 3). In unstructured environments predation increased diversity and especially so under the antibiotic treatment. In contrast, in structured environments, predation reduced diversity in the absence of the antibiotic but increased it with antibiotic added (both: spatial structure × predation: *P* < 0.001).

### Widespread, ecology-dependent transfer of resistance plasmid

We used emulsion, paired isolation, and concatenation PCR (epicPCR)^16^ for detection of plasmid recipient strains under experimental regimes. The method is based on linking a phylogenetic marker (here, 16S rRNA gene) with another gene of interest (here, kanamycin resistance gene *aphA* serving as RP4 plasmid marker) at single cell resolution, allowing us to identify RP4 plasmid recipient strains directly from multispecies experimental samples.

**Fig. 4:**
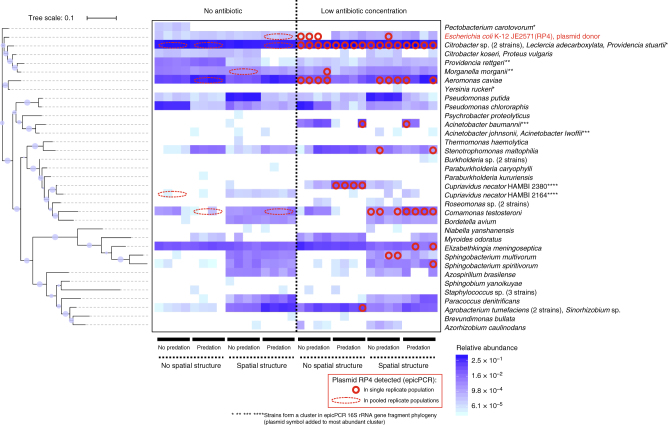
Relative abundance of strains and identity of strains in which plasmid RP4 was detected using epicPCR at the end-point of 40-day microcosm experiment. Based on the results from preliminary phenotypic screening, plasmid detection was performed separately for four replicate populations for the antibiotic treatment subtreatments (red circles) and for a pool of the four replicate populations for antibiotic-free subtreatments (red dashed line ovals). Strains are clustered in the heat map according to the phylogenetic tree on the left. The symbol size in the tree represents bootstrap values in the range 0.23–0.999. The plasmid donor strain *Escherichia coli* K-12 JE2571(RP4), present at low abundance, occasionally appears as negative for the plasmid; this is not taken to indicate absence of the plasmid but rather failure to exceed the conservative cutoff value used for plasmid presence in the epicPCR analysis pipeline to minimize the risk of false positives

**Fig. 5:**
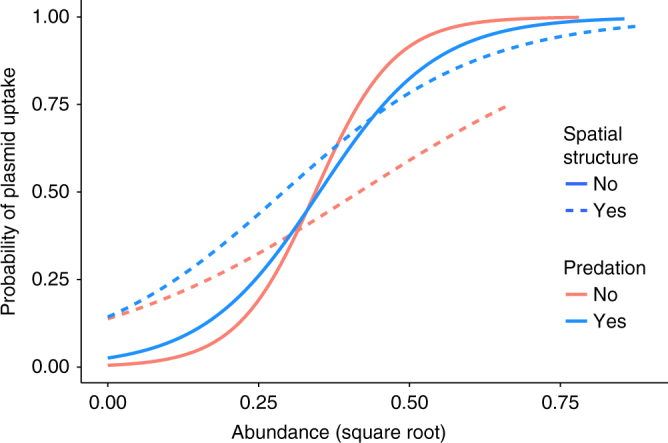
Logistic regression of the probability of acquiring the RP4 plasmid and taxon abundance under experimental regimes. Increasing strain abundance increases the probability of plasmid uptake (*P* < 0.0001). There is a significant interaction between abundance and spatial structuring (*P* = 0.0026): spatial structure reduces the influence of abundance. The effect of predation is not statistically significant (*P* = 0.26)

Widespread plasmid transfer was observed across treatments, with over twice as many plasmid recipient strains under low antibiotic concentration compared with no antibiotic (logistic regression, *P* = 0.013; Fig. 4). Altogether, across treatments, plasmid recipients were detected among approximately 35% of the total taxa present in communities. However, the plasmid recipient taxa depended on the treatments, which could explain 71% of variation in between-sample plasmid acquisition profiles (MRM for plasmid profiles with Sørensen dissimilarity: spatial structure *R*^2^ = 0.33, *P* < 0.001; predation *R*^2^ = 0.25, *P* < 0.001; spatial structure × predation *R*^2^ = 0.13, *P* = 0.004; Fig. 4). It is unlikely that the probability of receiving a plasmid was dependent on the original MIC values of the strains, since plasmid recipients exhibited a wide range of antibiotic MIC values, in many cases several-fold higher than the treatment concentration (Fig. 4, Supplementary Table 3). The probability of acquiring the plasmid was strongly dependent on strain abundance (*P* < 0.0001; Fig. 5). However, spatial structure weakened this link (*P* = 0.0011), such that plasmid transfer was more likely to occur to low-abundance strains in spatially structured environments (*P*_interaction_ *=* 0.0038). Strain abundance co-varied with taxonomical distance from the plasmid donor strain, belonging to the family Enterobacteriaceae, such that across treatments plasmid transfer occurred frequently to abundant, closely related Enterobacteriaceae strains, but with spatial structures more frequently to low-abundance, taxonomically distant strains (Supplementary Fig. 2).

### Population size

During the experiment, we tracked bacterial and ciliate population size over time via optical density and light microscopy based methods, respectively. While it is rather unsurprising that predation reduces the abundance of bacteria (generalized estimation equation model; *P* < 0.0001), it is less trivial that spatial structuring acts to increase microbial optical density (*P* < 0.0001), especially in the absence of predation (Supplementary Fig. 3a). Interestingly, antibiotics only had an effect in the absence of both spatial structure and predation, resulting in lower microbial density (Supplementary Fig. 3a). Predator abundance was elevated under sub-MIC of antibiotic (*P* < 0.001) and lowered in the presence of spatial structure (*P* < 0.001), with only marginally significant interaction between the treatments (*P*_interaction_ = 0.06) (Supplementary Fig. 3b).

## Discussion

We investigated the interactive effects of a low antibiotic concentration, spatial structure, and predation on an experimental microbial community of 62 strains, including the potential for spread of an antibiotic resistance plasmid. While we found that a low antibiotic concentration altered bacterial community composition and diversity, this effect depended on interactions with spatial structure and predation. This same phenomenon was apparent in horizontal transfer of a conjugative antibiotic resistance plasmid. The antibiotic increased the number of plasmid recipient taxa, but recipient profiles and the association between likelihood of receiving the plasmid and strain abundance were altered by presence/absence of spatial structure and/or predation. These results suggest that determining relevant ecological (biotic and abiotic) factors is critical for understanding the effects of low antibiotic concentrations on microbial communities and horizontal gene transfer potential.

The biotic and abiotic factors investigated in this study strongly determined the community-level effects of antibiotic. As predicted, antibiotic reduced diversity^4^, and this effect was reduced by antibiotic in combination with diversity-enhancing predation^17,29^. However, unexpectedly, diversity was higher in the combined treatment compared to the antibiotic-free environment or predation alone. Synergistic enhancement of a bacterial lifestyle or environment promoting diversity could result, for example, from improved cell aggregation associated both with aminoglycoside antibiotics^30^ (here, kanamycin) and protozoan predation^31^. Moreover, in line with our prediction, the diversity-reducing effect of antibiotic was lost in the presence of spatial structures. Bacteria attached to biofilms are 10–1000-fold less susceptible to antibiotics compared with planktonic cultures^32^, which is likely to account for this observation.

Compared with previous laboratory studies^7–10,12,33–35^, this study utilizes for the first time to our knowledge a complex multispecies community setting to investigate ecological mechanisms of antibiotic resistance. Our findings show a large number of plasmid recipient taxa across treatments, demonstrating the ability of a broad host range conjugative plasmid to infect a Gram-negative dominated community despite low competitive ability of its host. This is in line with recent studies showing high conjugation efficiency^13^ and plasmid maintenance^12,36^ in different scenarios regardless of the presence of positive selection. Importantly, plasmid recipient profiles differed between ecological scenarios, which supports the hypothesis that gene exchange networks are strongly structured by ecology^37^. Here the number of recipient taxa was also moderately elevated under sub-MIC antibiotics. This could be caused by the antibiotic selecting for a more diverse community or a community including more taxa within the plasmid host range. Alternatively and more likely, since diversity and community composition display strong variability across the antibiotic-free and antibiotic subtreatments, an increased number of recipient taxa can indicate an increased prevalence of the plasmid. Recent evidence suggests that such increase in plasmid prevalence under positive selection is generally caused by increased selection of transconjugants rather than by an increase in plasmid transfer events^10,11^. Increased plasmid spread and/or prevalence could also be an indirect result of altered community composition under antibiotic. For example, strain abundance is positively associated with growth rate in the antibiotic treatment, and higher growth rates of plasmid recipient strains might increase their ability to maintain high rates of plasmid conjugation in the system. There is also the possibility for source-sink dynamics whereby an abundant plasmid-harboring taxon, such as the Enterobacteriaceae strain cluster here, allows for transient plasmid maintenance in unsuitable hosts^38^, although this cannot be inferred from our data.

Furthermore, plasmid transfer most frequently occurred to abundant strains closely related to the plasmid donor rather than being driven by low initial MIC values of recipients expected to benefit most from plasmid transfer. Interestingly, in a spatially structured environment, the connection between the likelihood of receiving the plasmid and strain abundance was decoupled, such that the plasmid was more likely to be transmitted to low-abundance strains. This could be facilitated by improved mating pair formation in biofilms^23–25,39^. The abundance-dependency of plasmid transfer and increased number of recipients under antibiotic might, in part, be accounted for by the potential inability of the epicPCR method to detect the plasmid in low abundance strains. However, this is unlikely to explain the difference between the structured and unstructured environments. Further studies are needed to investigate the applicability of our observations to different antimicrobial agents and concentrations as well as their relevance with regard to natural, diverse bacterial communities.

## Methods

### Strains and culture conditions

To test for the effect of sub-MIC of antibiotic, spatial heterogeneity and predation on community structure and plasmid spread in a multispecies bacterial community, we constructed an artificial community consisting of 62 bacterial strains, named HMC62 (Supplementary Table 3). The strains were selected based on potential relevance for antibiotic resistance evolution via frequent occurrence in human-impacted environments or the gastrointestinal tract of mammals, including several opportunistic pathogens and members of the family Enterobacteriaceae. As plasmid donor strain, we used *Escherichia coli* K-12 strain JE2571(RP4)^40^, carrying the broad-host-range conjugative plasmid RP4 (incompatibility group IncP-1) encoding for kanamycin, ampicillin and tetracycline resistance. As predator species, we used the ciliated protist *Tetrahymena thermophila* CCAP 1630/1U frequently used in microcosm studies^41–43^.

We followed established protocols for microcosm experiments used with similar microbial model systems^42,44,45^. In order to enhance the maintenance of species diversity, we constructed a complex culture medium containing a variety of carbon sources. The medium contained M9 salts and King’s B (KB) nutrients at a 1% concentration compared with full-strength medium (concentrations used: 0.2 g peptone number 3 and 0.1 ml of 85% glycerol in 1 l of dH_2_O), and 0.2 g l^–1^ protozoan pellets (Carolina Biological Supply Co., Burlington, USA). Protozoan pellets were prepared by dissolving in dH_2_O, bringing to boil and filtering through 40 µm to remove particulate matter. All media and microcosm vials were sterilized by autoclaving prior to use and were kept at 28 °C (±0.1 °C) without shaking during the experiments.

### Determining strain traits

The experimental strains possess a wide range of minimum inhibitory concentrations (MICs) of the antibiotic kanamycin (Supplementary Table 3). Kanamycin MIC was determined for each strain after culturing one clone of each strain in PPY medium for 48 h by spread-plating 100 µl on a 50% PPY agar plate using Liofilchem^®^ MIC test strips (Liofilchem, Italy) according to the manufacturer’s instructions. Note that the test strip MIC values on agar plates are systematically lower compared to liquid cultures while remaining comparable between samples. This likely explains the survival of several low-MIC strains in the experiment under apparently above-MIC antibiotic exposure. To control for the absence of the plasmid RP4 or other IncP-1 plasmids in non-donor strains, we performed PCR for the plasmid-encoded kanamycin resistance gene *aphA*, and replication initiation protein *trfA*, respectively, using previously published primers^46,47^ with the plasmid donor as positive control (detailed protocol in Supplementary Information).

To determine the carrying capacity and biofilm formation ability of each strain, strains were cultured in experimental medium for 96 h using the Bioscreen C well-plate reader (Labsystems Oy, Helsinki, Finland) to measure optical density (OD) at 420–580 nm with a wideband filter at 5 min intervals. The carrying capacity of the strain was interpreted as mean OD during the last 2 h of measurement. Subsequently, a biofilm-formation assay was performed as described previously^48,49^. Briefly, after the OD readings, 1% of crystal violet was transferred to each well, and after 10 min, wells were rinsed three times with dH_2_O. Thereafter, 450 µl of 96% ethanol was added to wells to dissolve the crystal violet attached to biofilm. OD measurement was performed for 24 h as described above, and biofilm formation ability was estimated as the amount of dissolved crystal violet obtained (mean OD during the last 2 h of measurement).

### Microcosm experiment

To test for the effect of sub-MIC of antibiotic, spatial heterogeneity and predation on bacterial communities and plasmid transfer, we conducted a full factorial 40-day-long microcosm experiment. First, to test for community-level effects of sub-MIC antibiotic level and to select for plasmid accessory traits, we had an antibiotic treatment with 0 or 2 µg ml^−1^ kanamycin (2 µg ml^−1^ represents approximately 6% of the mean MIC value of strains). Second, to test for the effect of a spatially structured environment, we had a treatment with absence/presence of glass beads in culture vials creating a dual-phase (liquid phase and glass bead phase) structured environment. Spatial structure vials were prepared by adding 5 g of 0.1–0.2 mm glass beads (SiLibeads^®^ Glass beads Type S, Sigmund Lindner, Germany) that had been rinsed with boiling dH_2_O and dried prior to use. Third, to test for the effect of trophic (here, predator-prey) interactions, we had a ciliate presence/absence treatment.

Prior to the experiment, individual colonies of strains were cultured separately in PPY medium for 48 h. Subsequently, the plasmid donor strain *E*. *coli* JE2571(RP4) and all other strains pooled together (200 µl/strain) were spinned down and resuspended in M9 salts at OD 0.2 measured at 600 nm wavelength (UV-1800 spectrophotometer, Shimadzu, Japan). These bacterial suspensions were used to start the experiment as well as frozen in 28% glycerol and kept at –80 °C for later use as an immigration stock. The protozoa were prepared by spinning cells down and resuspending in M9 salts overnight to starve cells prior to starting the experiment. The treatments were started by adding 150 µl of the bacterial stock and 150 µl of the plasmid donor strain in M9 salts to culture vials containing 5.7 ml of culture medium. *T*. *thermophila* was added at an initial density of 1000 cells per ml to ciliate treatments. All treatments (antibiotic, spatial structure and predator) were replicated four times in 25 ml glass vials, which were kept unshaken, containing a final volume of 6 ml medium. Every 96 h, 500 µl each of freeze-stored bacterial stock and plasmid donor strain were mixed and suspended in a final volume of 10 ml M9 salts, and 2.5% (150 µl) of each culture together with a 2.5% (150 µl) immigration of the bacterial stock–plasmid donor strain mixture (to prevent loss of plasmid donor strain and community diversity with extended culturing) was transferred to a new vial containing 5.7 ml of fresh culture medium. The previous culture vial was vortexed briefly prior to transfer to ensure transfer of a representative subset of the community and to detach cells from the glass beads. A coarse estimate of total bacterial abundance was obtained with the OD-based method described above (glass bead effect was removed from OD values of spatial structure samples), and ciliate cell densities were enumerated directly from live samples using a compound microscope (Zeiss Axioskop 2 plus, Oberkochen, Germany). The relationship between OD and viable cell counts is shown in Supplementary Fig. 4. A 1.0 ml subsample was frozen in 28% glycerol and kept at –80 °C for later analysis.

### Determining spread of RP4 plasmid

To obtain a coarse estimate of the presence of plasmid in experimental treatments, 100 µl of experimental culture from each vial was spread-plated in the end-point (day 40) of the experiment on 50% PPY agar plates containing 150 µg ml^–1^ ampicillin, 25 µg ml^–1^ kanamycin, and 20 µg ml^–1^ tetracycline, and plates were monitored for bacterial growth after culturing for 96 h. Because colonies were only observed in the antibiotic treatment, to examine the spread of the plasmid RP4 during the experiment, emulsion, paired isolation and concatenation PCR (epicPCR^16^) was performed for the antibiotic treatment at subtreatment replicate level resolution and for the antibiotic-free treatment by pooling together samples from each of four subtreatment replicates.

The epicPCR procedure was performed as described previously in detail in ref. ^16^ and the associated protocol in Protocol Exhange (hereafter, PE; doi:10.1038/protex.2015.094), using the *aphA* gene to design fusion and nested primers for the plasmid RP4. The primers used are listed in Supplementary Table 4. Briefly, cells were pretreated by spinning down 0.5 ml of freeze-stored sample (for pooled antibiotic-free subtreatment samples, 150 µl each from four replicates) and resuspending in 30 µl of nuclease-free water. Cells were trapped in polyacrylamide beads as described in PE, except that the polymerization mixture consisted of 30 µl of suspended cells, 100 µl of nuclease-free water, 100 µl of 30% Bis-acrylamide (Bio-Rad Laboratories, California, USA) and 25 µl of 10% ammonium persulfate. To confirm min. 100:1 bead-to-cell ratio (max. one cell per bead assuming Poisson distribution), beads containing SYBR-stained bacterial cells were visually inspected under a compound microscope (Zeiss Axioskop 2 plus, Oberkochen, Germany). Fusion PCR was performed with 46.5 µl of sample beads, 16 U of Phusion® Hot Start Flex DNA Polymerase (New England Biolabs, Massachusetts, USA), 0.1 µM each of F1 and R2 primers, 0.01 µM of R1-F2′ (linker) primer, 1 mM MgCl_2_, and 250 μM of dNTPs at a final volume of 100 µl of 1× Phusion HF buffer. The PCR mix was emulsified with ABIL emulsion as described in PE, and PCR was performed with the following cycling conditions: initial temperature 80 °C, 94 °C for 30 s, and 32 cycles of 94 °C for 5 s, 52 °C for 30 s, and 72 °C for 30 s, with a final extension at 72 °C for 5 min. The ABIL emulsion was broken with ethyl ether and PCR products were purified using the Agencourt^®^ AMPure^®^ XP PCR purification kit (Beckman Coulter, Brea, CA, USA) as described in PE. Nested/blocking PCR was performed with 2 µl of purified fusion PCR product, 0.5 U of Phusion High-Fidelity DNA Polymerase (Thermo Fisher Scientific, Waltham, MA, USA), 0.3 µM each of the F3 and R3 primers, 3.2 µM each of the blockF and blockR primers, and 200 µM of dNTPs at a final volume of 25 µl of 1× Phusion HF Buffer. The cycling conditions were as follows: 98 °C for 30 s, and 39 cycles of 98 °C for 10 s, 52 °C for 30 s, and 72 °C for 30 s, with a final extension at 72 °C for 5 min. The fused, nested products underwent a final, short 17-cycle amplification to add flow-cell compatible Illumina adapters as described in PE, and products purified using the Agencourt^®^ AMPure^®^ XP PCR purification kit were submitted for paired end Illumina MiSeq sequencing (2 × 300 bp).

### DNA extraction and sequencing

To determine community composition in the microcosm experiment, 16S rRNA amplicons were shotgun-sequenced from all replicates and treatments at days 8 (2 transfers), 24 (6 transfers), and 40 (10 transfers; end-point) in the experiment (*N* = 96). For this, DNA was extracted from 0.5 ml of freeze-stored experimental samples using PowerWater^®^ DNA Isolation Kit (MoBio, Carlsbad, CA, USA). Samples were spinned down, 400 µl of supernatant was removed, and the remaining 100 µl was transferred to PowerWater^®^ Bead Tubes, after which extraction was performed according to the manufacturer’s instructions. DNA concentrations were measured using the Qubit^®^ 3.0 fluorometer (Thermo Fisher Scientific, Waltham, MA, USA).

Paired-end sequencing (2 × 300 bp) was performed using the Illumina MiSeq platform at the Institute for Molecular Medicine Finland. The 16S rRNA V3 and V4 region was amplified using Phusion High Fidelity PCR Master Mix (Thermo Fisher Scientific, Waltham, MA, USA). Reactions were done as multiplex PCR reactions with two 16S rRNA gene primers carrying Illumina adapter tails (forward primer 341 F 5′-CCTACGGGAGGCAGCAG-3′ and reverse 805 R 5′-GACTACHVGGGTATCTAATCC-3′^50^ and two Illumina P5/P7 index primers (every sample had their own unique combination). PCR amplification was performed in a volume of 20 µl containing approximately 20 ng of sample DNA, 1 µl (5 µM) of each locus-specific primer (final concentration 0.25 µM), 1.5 µl (5 µM) of each index primer (final concentration 0.375 µM), and 10 µl of 2 × Phusion High-Fidelity PCR Master Mix, and the reaction mix was brought to a final volume with laboratory grade water. The cycling conditions were as follows: 98 °C for 30 s, 27 cycles of 98 °C for 10 s, 62 °C for 30 s, and 72 °C for 15 s, with a final extension at 72 °C for 10 min, followed by hold at 10 °C. After PCR, random samples were measured with LabChip GX Touch HT DNA High Sensitivity Reagent Kit (Perkin Elmer, Waltham, MA, USA) to check that the PCR was successful with the correct product size. Samples were pooled together in equal volumes and purified with Agencourt^®^ AMPure^®^ XP beads (Beckman Coulter, Brea, CA, USA) twice using 0.8× volume of beads compared to the sample pool volume (40 µl). The ready amplicon library was diluted to 1:10 and quantified with the Agilent 2100 Bioanalyzer High Sensitivity DNA Analysis Kit (Agilent Genomics, Santa Clara, CA, USA). The 16S rRNA gene amplicon pool and epicPCR products were sequenced in one flow cell with the Illumina MiSeq System using the Illumina MiSeq Reagent Kit v3 600 cycles kit (Illumina, San Diego, CA, USA). We obtained a total of 20.9 Gb of combined sequence data from 16S rRNA and epicPCR amplicons.

In addition, we also sequenced the 16S rRNA gene (near full length) of all 62 experimental strains to be used as a reference database. The 16S rRNA gene was amplified using colonic PCR with the universal primers pA and pH´^51^. To obtain template DNA, individual colonies were suspended in 50 µl of autoclaved dH_2_O and boiled at 100 °C for 10 min. PCR was performed with 2 µl of boiled colony, 1 U of DyNAzyme II DNA Polymerase (Thermo Fisher Scientific, Waltham, MA, USA), 0.2 µM of each primer, and 200 µM of dNTPs in a final volume of 50 µl of 1× Optimized DyNAzyme buffer. The cycling conditions were as follows: 98 °C for 5 min (to improve cell lysis), 98 °C for 30 s, and 25 cycles of 98 °C for 10 s, 60 °C for 30 s, and 72 °C for 45 s, with a final extension at 72 °C for 5 min. PCR products were Sanger sequenced at the Institute of Biotechnology (University of Helsinki, Finland).

### Sequence analyses

To construct reference sequence databases, near full-length 16S rRNA gene sequences were assembled from Sanger sequencing chromatograms using Pregap4 and Gap4 in the Staden Package^52^. Reads were aligned with ClustalW, and the 16S rRNA gene fragments corresponding to the regions sequenced from epicPCR (V4) and whole-community samples (V3–V4) were extracted using MEGA7^53^. To obtain centroid sequences for use as reference databases, the two sequence sets were clustered using the USEARCH v8.0^54^ -cluster_fast command with -id 0.97 and -centroids parameters. The 62 strains formed 37 and 47 clusters with the epicPCR and whole-community amplicon regions of the 16S rRNA gene, respectively. The phylogenetic tree in Fig. 4 was constructed by aligning whole-community 16S rRNA gene amplicon centroid sequences with PyNAST^55^ and creating a tree with FastTree 2.1.3^56^ using default parameters, with visualization in iTOL^57^.

With MiSeq sequencing reads, sequencing adapters were removed using Cutadapt v1.12^58^ and paired-end reads were merged using PEAR v0.9.8^59^ with default settings. Reads were quality filtered with the USEARCH v8.0 -fastq_filter command with -fastq_maxee 1.0 and -fastq_minlen 400 (300 for epicPCR reads) parameters. The presence of fusion products in epicPCR reads was confirmed by searching against the nr nucleotide database (NCBI, National Center for Biotechnology Information) using the megaBLAST^60^ algorithm with default parameters. To retain only the 16S rRNA gene fragment in epicPCR reads, the RP4 plasmid fragment was removed by splitting reads using the Python tool epride (https://github.com/manutamminen/epride, last accessed January 2017). Following splitting libraries based on barcodes and concatenation of fasta files, which was performed separately for epicPCR and whole-community reads, unique sequences were identified with the VSEARCH v2.4.2^61^ --derep_fulllength command. Operational taxonomic units (OTUs) were clustered and reads were mapped to reference databases with USEARCH v9.2, using the -cluster_otus command with -minsize 2 parameter and the -usearch_global command with -id 0.97 parameter, respectively. Out of the epicPCR and whole-community reads, 97.1 ± 1.4% and 98.5 ± 1.5%, respectively, mapped to the reference databases. After quality filtering and mapping, we had a total of 2,051,888 epicPCR and 6,286,634 whole-community reads which were used for downstream analyses.

Due to the presence of several amplification steps in the epicPCR protocol that may cause strong PCR bias, the epicPCR reads mapped to the reference database were treated as on/off rather than quantitative data. To decrease the risk of false positives that could result, for example, from the occasional presence of two or more cells from different taxa in the same droplet, a conservative per sample cutoff value of 0.03 was used for presence (i.e. taxa with ≥3.0% relative abundance were coded as 1 for presence of plasmid and other taxa with <3% abundance as 0 for absence of plasmid). To prevent underestimation of plasmid recipients in treatments without antibiotic, four-fold dilution of each individual sample during pooling of antibiotic-free subtreatment samples was accounted for by using a four-fold lower cutoff value (0.0075). When the plasmid was detected in a taxon clustering differently with the epicPCR and whole-community 16S rRNA gene regions, the corresponding whole-community cluster was assigned based on the presence or absence of epicPCR cluster members in the community (Fig. 4).

### Statistical analyses

*Time series analysis*: We analyzed experimental time series of microbial optical density and ciliate predator density in samples. Prior to the analysis, the first two time points were discarded as transients. In the case of Microbial OD (log-transformed), we used a generalized least squares model (gls), as implemented in the nlme^62^ package in R^63^. We assumed AR1 residual correlation structure within replicates and additionally specified a residual variance structure dependent on the experimental treatments. Predator densities were analyzed with a generalized estimation equation model (gee), as implemented in the geepack package^64^, assuming AR1 residual correlation within replicates.

*Community level analysis*: Microbial community composition was characterized using Bray and Curtis dissimilarity on square root-transformed relative abundances. Variation in dissimilarity across experimental treatments was analyzed with permutational multivariate regression on distance matrices (MRM), as implemented in the vegan package^65^. For visualization, we ran a random forest classification of the community data (as implemented in the randomForest package^66^ and used the predicted proximities to produce a non-metric multidimensional scaling (NMDS) ordination, using the hybrid model (monoMDS function in the vegan package). Community diversity was quantified using the Shannon entropy (diversity of first degree)^67^.

*Strain level analysis*: To analyze patterns in strain occurrence, we utilize a framework of hierarchical modeling of strain communities (HMSC)^28^, which is able to account for the joint dispersion of strain due to phylogenic correlation, strain traits, and random effects. We fitted the model using presence/absence data, strain traits (MIC value, growth rate, and biofilm formation), phylogenetic correlation (cophenetic correlation calculated from the phylogenetic tree), and random effects (sampling unit, replicate, and day). For the Bayesian estimation, we used 20,000 iterations with 10,000 burn-in. The HMSC method allows for estimating how strain traits are associated with explanatory variables, resembling a 4th corner approach^68^. As a comparison, we also ran a 4th corner-type analysis (traitglm) implemented in the mvabund package^69^, which allows analysis of abundance data (not currently implemented in the R-version of HMSC).

*Patterns in plasmid uptake*: In order to analyze co-uptake patterns of the plasmid, we calculated the Sørensen dissimilarity between samples. This matrix was analyzed with MRM as above. We also analyzed the plasmid acquisition probability of strains using logistic regression. The model was fitted using glmmPQL function in the MASS package^70^, setting strain as a random factor.

### Data availability

All raw sequence reads have been deposited in the National Center for Biotechnology Information (NCBI) Sequence Read Archive (SRA) under the BioProject Accession Number PRJNA393619. Near-full-length 16S rRNA gene sequences for all 62 experimental strains have been deposited in the European Nucleotide Archive (ENA) under the Accession Number PRJEB21728. From downstream analyses, (1) a strain abundance table based on whole-community 16S rRNA amplicon analysis and (2) RP4 plasmid presence/absence data table based on epicPCR amplicon analysis are available via Dryad: 10.5061/dryad.5756sg0.

## Electronic supplementary material

Supplementary Information
