# Supplementary material for: Ecology determines how low antibiotic concentration impacts community composition and horizontal transfer of resistance genes
Source: Commun Biol. 2018 Apr 19;1:35. doi: 10.1038/s42003-018-0041-7 (PMC6123812; doi:10.1038/s42003-018-0041-7)
Supplement: Supplementary file 1 — Supplementary Information [file 42003_2018_41_MOESM1_ESM.pdf]

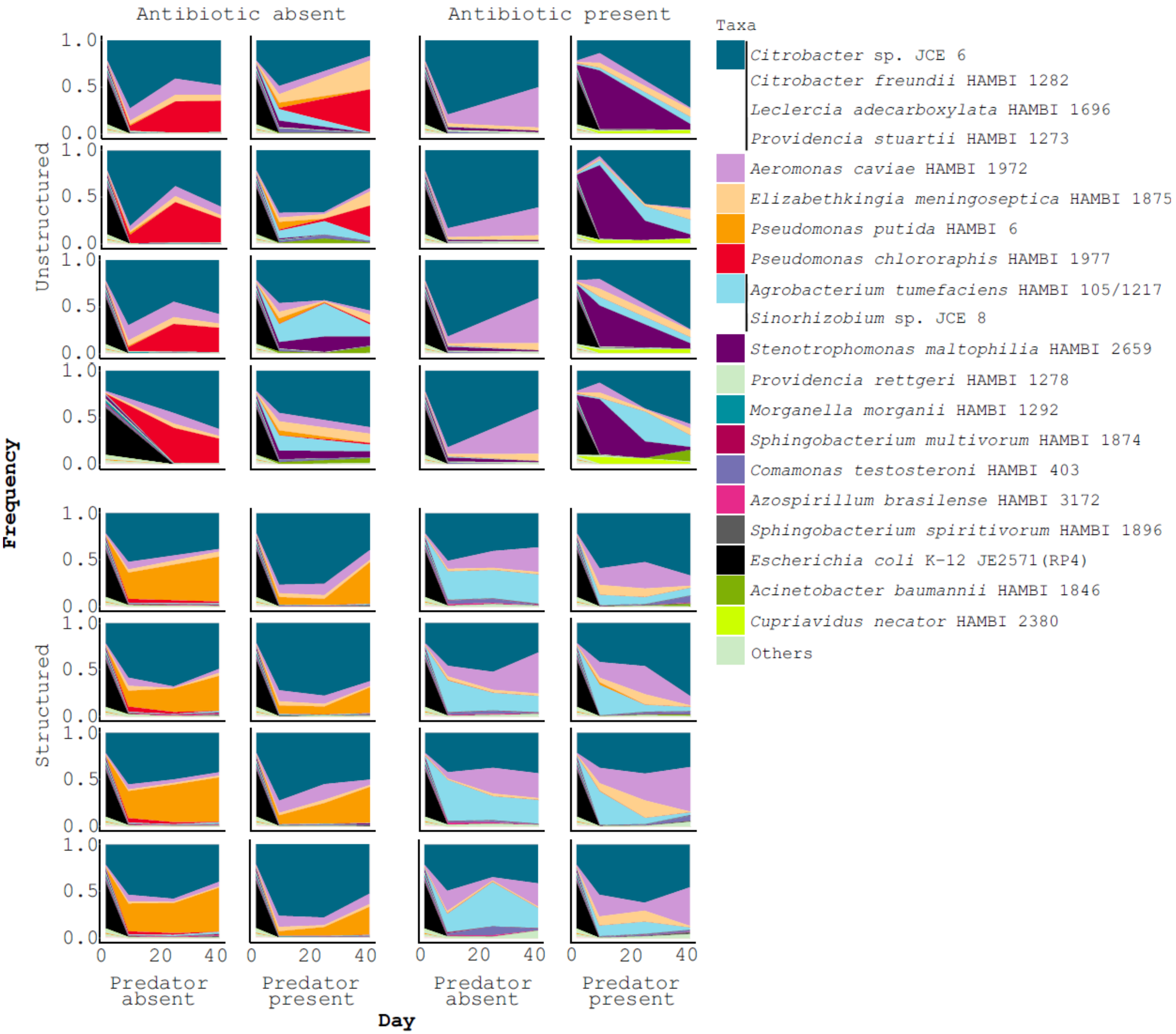

Supplementary Figure 1. Taxa across time points and treatments in 40-day microcosm experiment.

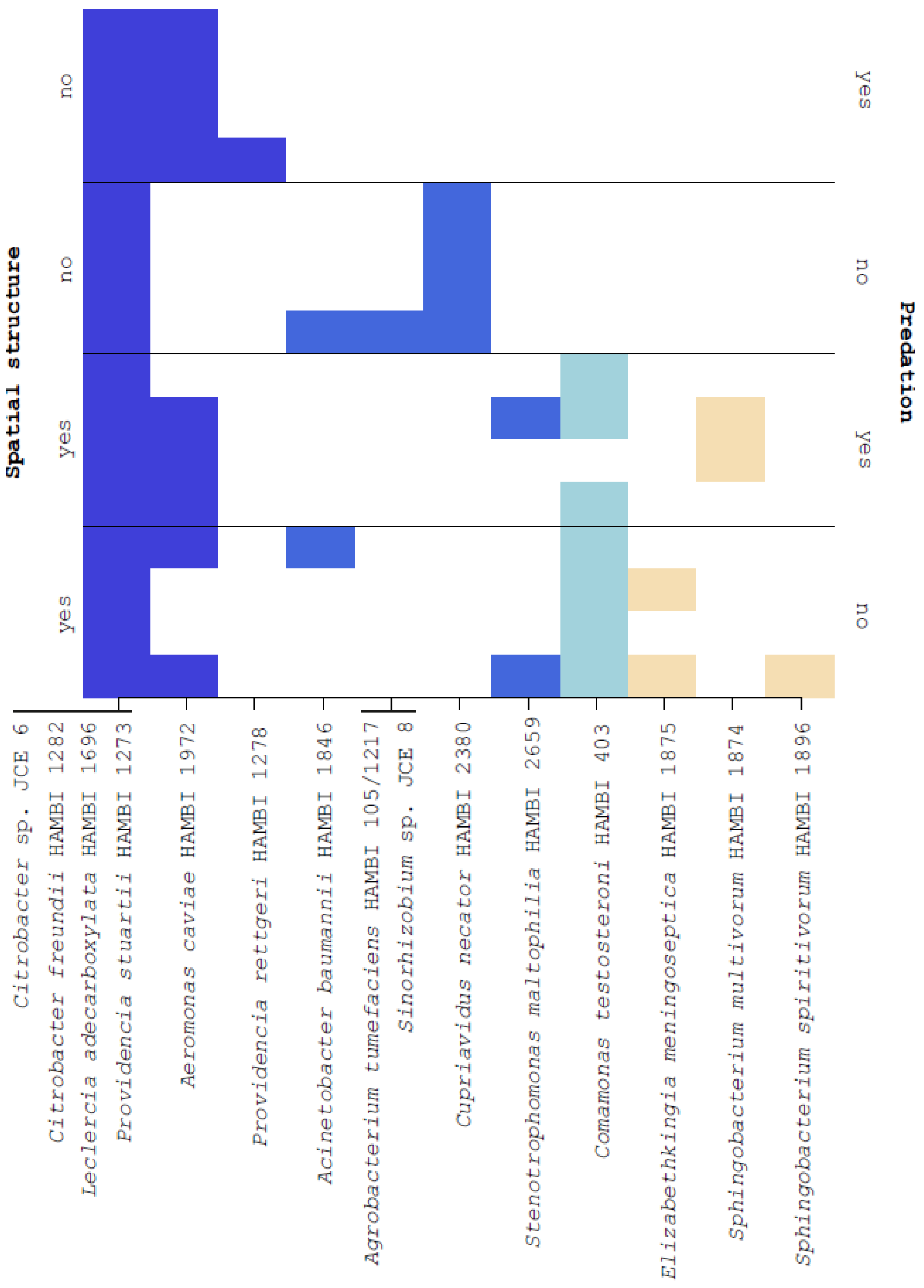

Supplementary Figure 2. Taxonomic distance of plasmid recipients from plasmid donor strain *Escherichia coli* JE2571(RP4) in the antibiotic treatment subtreatments at the end-point of 40-day microcosm experiment. The distance is scaled from blue, indicating low distance, to yellow, indicating high distance.

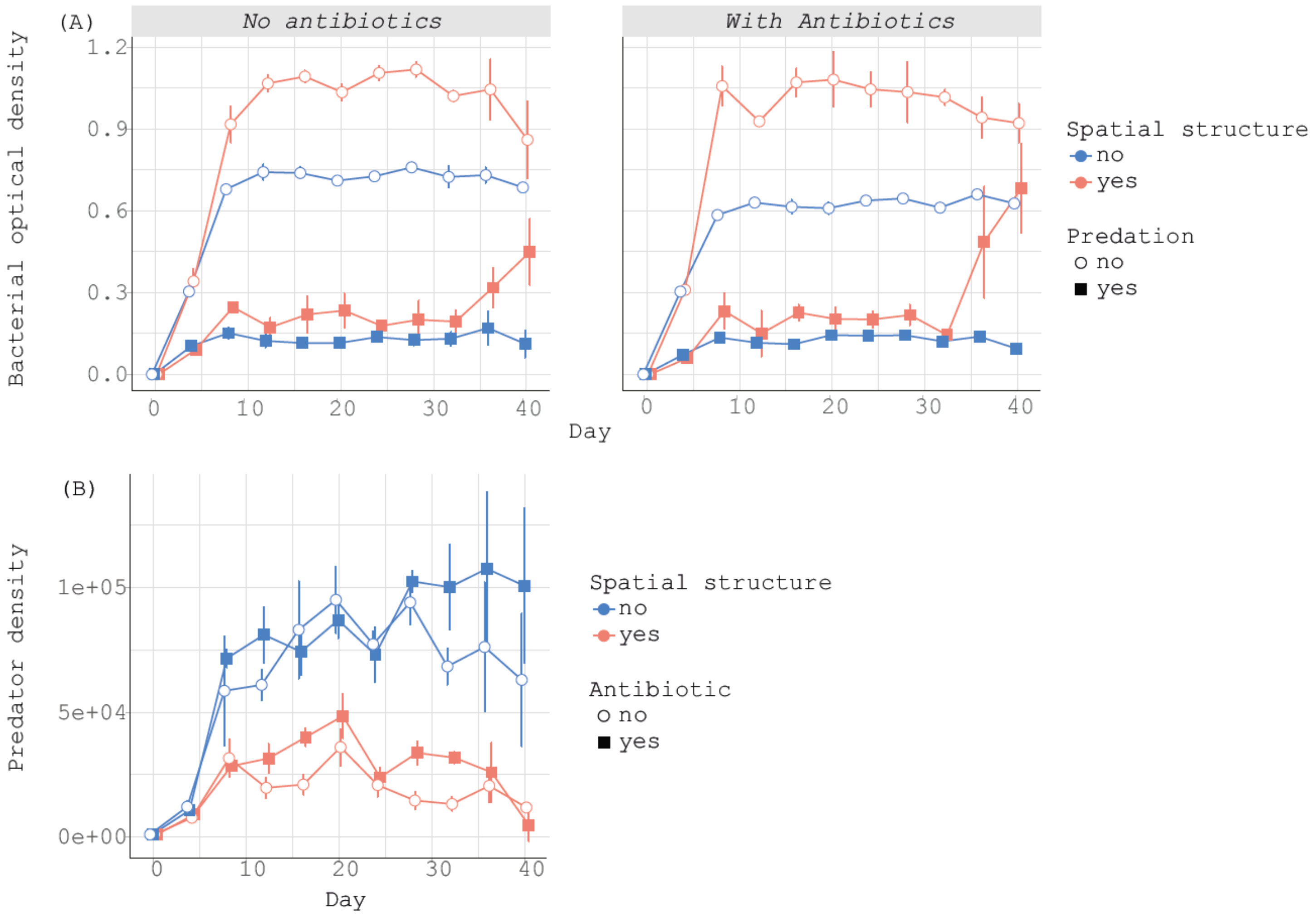

Supplementary Figure 3. (A) Density of bacteria and (B) ciliate predator across time points and treatments in 40-day microcosm experiment (mean ± SD; N = 4 per treatment combination).

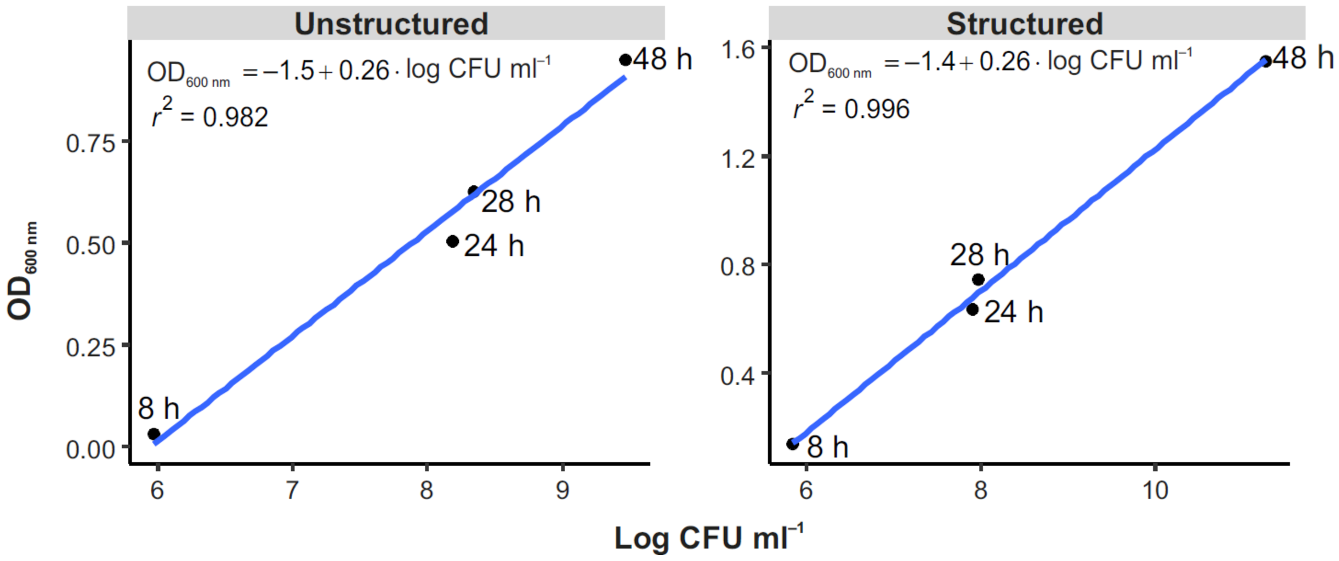

Supplementary Figure 4. Relationship between optical density (OD) at 600 nm and colony forming units (CFU) in the experimental conditions (medium, inoculum, temperature, treatment). The data points are averages of three replicated 48 h growth experiments in each environment (unstructured/structured).

Supplementary Table 1. MRM model of between-sample dissimilarity. Based on Bray&Curtis dissimilarity calculated over sqrt-transformed relative microbial abundances. Statistical significance was tested with 999 random permutations.

Model terms	Df	MS	*F*	R^2	*P*
Day	1	0.17	7.82	0.03	0.001
Antibiotic	1	1.15	53.73	0.19	0.001
Spatial structure	1	1.18	55.04	0.19	0.001
Predation	1	0.44	20.71	0.07	0.001
Antibiotic × Space	1	0.55	25.64	0.09	0.001
Antibiotic × Predation	1	0.10	4.51	0.02	0.003
Space × Predation	1	0.76	35.54	0.13	0.001
Antibiotic × Space × Predation	1	0.07	3.48	0.01	0.003
Residuals	77	0.02		0.27	

Supplementary Table 2. Trait by treatment interaction estimates from the HMSC model and additionally for abundances using traitglm in the mvabund package.

Method	Treatment	MIC value	Growth rate	Biofilm formation
HMSC	Antibiotic	0.15	0.07	0.19
	Predation	0.18	0.13	0.06
	Spatial structure	**0.44**	**0.43**	0.18
traitglm	Antibiotic	**0.25**	**0.26**	0.15
	Predation	**0.33**	0.06	0.07
	Spatial structure	**0.23**	**0.42**	0.11

Supplementary Table 3. Composition of HAMBI Mock Community (HMC) 62 created for this study.

**Strain**	**Kanamycin MIC (μg ml^{-1})**
Acinetobacter baumannii HAMBI 1846[a]	0.50
Acinetobacter johnsonii HAMBI 1969[a]	0.50
Acinetobacter lwoffii HAMBI 97[a]	0.38
Aeromonas caviae HAMBI 1972[a]	1.0
Aeromonas hydrophila HAMBI 1847[a]	1.5
Agrobacterium tumefaciens HAMBI 105[a]	4.0
Agrobacterium tumefaciens HAMBI 1217[a]	1.0
Azorhizobium caulinodans HAMBI 216[a]	2.0
Azospirillum brasilense HAMBI 3172[a]	2.0
Bacillus sp. JCE 7[b]	0.09
Bacillus sp. JCE 10[b]	0.50
Bordetella avium HAMBI 2160[a]	4.0
Brevundimonas bullata HAMBI 262[a]	0.75
Burkholderia sp. HAMBI 3356[a]	0.75
Burkholderia glumae HAMBI 2369[a]	1.0
Buttiauxella agrestis HAMBI 1320[a]	1.5
Chitinophaga filiformis HAMBI 1966[a]	> 256
Chitinophaga sancti HAMBI 1988[a]	64
Chryseobacterium sp. JCE 2[b]	128
Citrobacter sp. JCE 6[b]	1.5
Citrobacter amalonaticus HAMBI 1296[a]	1.5
Citrobacter freundii HAMBI 1282[a]	0.75
Citrobacter koseri HAMBI 1287[a]	1.5
Comamonas testosteroni HAMBI 403[a]	4.0
Cupriavidus necator HAMBI 2164[a]	4.0
Cupriavidus necator HAMBI 2380[a]	0.38
Elizabethkingia meningoseptica HAMBI 1875[a]	128
Escherichia coli K-12 JE2571(RP4)[31]	> 256
Hafnia alvei HAMBI 1279[a]	0.75
Kluyvera intermedia HAMBI 1299[a]	3.0
Leclercia adecarboxylata HAMBI 1696[a]	0.38
Leclercia sp. JCE 4[b]	0.75
Lelliottia amnigena HAMBI 1289[a]	0.38
Microvirga lotononidis HAMBI 3237[a]	8.0

Moraxella canis HAMBI 2792[a]	0.25
Morganella morganii HAMBI 1292[a]	0.50
Myroides odoratus HAMBI 1923[a]	192
Niabella yanshanensis HAMBI 3031[a]	> 256
Novosphingobium sp. JCE 5[b]	1.0
Paraburkholderia caryophylli HAMBI 2159[a]	1.0
Paraburkholderia kururiensis HAMBI 2494[a]	0.75
Paracoccus denitrificans HAMBI 2443[a]	0.75
Pectobacterium carotovorum HAMBI 1429[a]	0.75
Phyllobacterium myrsinacearum HAMBI 1992[a]	> 256
Proteus vulgaris HAMBI 91[a]	1.0
Providencia rettgeri HAMBI 1278[a]	1.5
Providencia stuartii HAMBI 1273[a]	1.5
Pseudomonas chlororaphis HAMBI 1977[a]	1.0
Pseudomonas putida HAMBI 6[a]	0.25
Psychrobacter proteolyticus HAMBI 2948[a]	< 0.02
Roseomonas sp. JCE 1[b]	128
Roseomonas gilardii HAMBI 2470[a]	2.0
Sinorhizobium sp. JCE 8[b]	192
Sphingobacterium multivorum HAMBI 1874[a]	> 256
Sphingobacterium spiritivorum HAMBI 1896[a]	> 256
Sphingobium yanoikuyae HAMBI 1842[a]	0.75
Staphylococcus sp. JCE 3[b]	1.5
Staphylococcus sp. JCE 9[b]	0.13
Staphylococcus sp. JCE 11[b]	0.25
Stenotrophomonas maltophilia HAMBI 2659[a]	16
Thermomonas haemolytica HAMBI 2467[a]	0.50
Yersinia ruckeri HAMBI 1298[a]	2.0

[a]HAMBI Culture Collection, University of Helsinki (UH).
[b]This study (strain available on request).

Supplementary Table 4. Primers used in epicPCR.

epicPCR code	Name	Sequence	Source
F1[a]	*aphA*_F1[*]	CGGTCTATCGGCTGCATAGCAAGTC	[1]
R1-F2'[b]	*aphA*_R1-F2'[*]	GWATTACCGCGGCKGCTGCAAGCCTCTCCTGAAGC GAAGGTTCG	This study[c]
R2[d]	1492R[*]	GGTTACCTTGTTACGACTT	[3]
F3[e]	*aphA*_F3[**]	ATTGCTGATCGATACCAAG	This study
R3[f]	PE16S_V4_E786_R[**]	CGGCATTCCTGCTGAACCGCTCTTCCGATCTGGACT ACHVGGGTWTCTAAT	[3]
blockF[g]	U519F-block10	TTTTTTTTTTCAGCMGCCGCGGTAATWC/3SpC3/	[3]
blockR[h]	U519R-block10	TTTTTTTTTTGWATTACCGCGGCKGCTG/3SpC3/	[3]

[*]Product size 955 bp with *aphA* gene and V4–V8 region of 16S rRNA gene.
[**]Product size 329 bp with *aphA* gene and V4 region of 16S rRNA gene.
[a]Functional target gene (*aphA* gene in PR4 plasmid) forward primer (fusion PCR).
[b]Functional target gene reverse primer with 16S rRNA gene forward primer overhang (fusion PCR linker primer).
[c]The *aphA* gene reverse primer from[1] is combined with the 16S rRNA overhang from[3].
[d]16S rRNA gene reverse primer (fusion PCR).
[e]Target gene nested primer (nested/blocking PCR).
[f]16S rRNA gene nested primer (nested/blocking PCR).
[g]Blocking forward primer (nested/blocking PCR).
[h]Blocking reverse primer (nested/blocking PCR).

Supplementary Methods

Testing for presence of RP4 or other IncP-1 plasmids in non-donor strains

To control for the absence of the plasmid RP4 in non-donor strains, we performed PCR for the RP4-encoded kanamycin resistance gene *aphA*, using the plasmid donor as positive control. For this, single colonies of strains were cultured in proteose peptone yeast extract (PPY) medium (20 g proteose peptone and 2.5 g yeast extract in 1 l dH_2O) for 48 h at 28 °C (± 0.1 °C) with constant shaking at 150 r.p.m. DNA was extracted from 0.5 ml of medium containing a pool of 5–7 strains using PowerWater® DNA Isolation Kit (MoBio, Carlsbad, CA, USA). Samples were spinned down, resuspended in 100 µl of autoclaved dH_2O, and transferred to PowerWater® Bead Tubes, after which extraction was performed according to the manufacturer's instructions. DNA concentrations were measured using the Qubit® 3.0 fluorometer (Thermo Fisher Scientific, Waltham, MA, USA). PCR was performed with 10–20 ng of template DNA, 1 U of Phusion Hot Start II DNA Polymerase (Thermo Fisher Scientific, Waltham, MA, USA), 0.2 µM each of the *aphA* primers CGGTCTATCGGCTGCATAGCAAGTC and CAAGCCTCTCCTGAAGCG-AAGGTTCG[1] (product size 627 bp), and 200 µM of dNTPs in a final volume of 50 µl of 1× 5X Phusion HF Buffer. The cycling conditions (2-step protocol) were as follows: 98 °C for 30 s, and 35 cycles of 98 °C for 10 s and 72 °C for 30 s, with a final extension at 72 °C for 5 min.

Using the same DNA pools, we also tested for the presence of other plasmids in the same incompatibility group, IncP-1, which might explain the lack of plasmid transfer to certain strains in the community. For this, we used previously published primers targeting the *trfA* gene encoding the plasmid replication initiation protein: for subgroups α, β and ε: TTCACSTTCTACGAGMTKTGCCA-GGAC and GWCAGCTTGCGGTACTTCTCCCA; for γ: TTCACTTTTTACGAGCTTTGCAGCGAC and GTCAGCTCGCGGTACTTCTCCCA; and for δ: TTCACGTTCTACGAGCTTTGCACAGAC and GACAGCTCGCGGTACTTTTCCCA (product size 281 bp).[2] Notably, a positive control strain was lacking for the γ subgroup primers. PCR was performed with 2–5 ng of template DNA, 1.25 U of DreamTaq DNA Polymerase (Thermo Fisher Scientific, Waltham, MA, USA), 0.5 µM each of the forward and reverse primers and 200 µM of dNTPs in a final volume of 50 µl of 1× 10X DreamTaq Buffer. The cycling conditions were as follows: initial denaturation 95 °C, 95 °C for 30 s, and 30 cycles of 95 °C for 30 s, 56 °C for 30 s, and 72 °C for 1 min, with a final extension at 72 °C for 5 min. All samples were negative for all primers except for the positive control strain.

Supplementary References

1 Jalasvuori, M., Friman, V. P., Nieminen, A., Bamford, J. K. & Buckling, A. Bacteriophage selection against a plasmid-encoded sex apparatus leads to the loss of antibiotic-resistance plasmids. *Biol Lett* **7**, 902–905 (2011).

2 Bahl, M. I., Burmolle, M., Meisner, A., Hansen, L. H. & Sorensen, S. J. All IncP-1 plasmid subgroups, including the novel epsilon subgroup, are prevalent in the influent of a Danish wastewater treatment plant. *Plasmid* **62**, 134–139 (2009).

3 Spencer, S. J. *et al.* Massively parallel sequencing of single cells by epicPCR links functional genes with phylogenetic markers. *ISME J* **10**, 427–436 (2016).
